# How Work–Family Conflict and Work–Family Facilitation Affect Employee Innovation: A Moderated Mediation Model of Emotions and Work Flexibility

**DOI:** 10.3389/fpsyg.2021.796201

**Published:** 2022-01-11

**Authors:** Zhicheng Wang, Xingyu Qiu, Yixing Jin, Xinyan Zhang

**Affiliations:** ^1^School of Business, Nanjing University, Nanjing, China; ^2^School of Business, Jiangxi University of Finance and Economics, Nanchang, China; ^3^School of Business, Jinan University, Guangzhou, China; ^4^School of Tourism, Huangshan University, Huangshan, China; ^5^School of Business, Chungnam National University, Daejeon, South Korea

**Keywords:** employee innovation, work–family conflict, work–family facilitation, negative emotions, positive emotions, work flexibility

## Abstract

This paper aims to verify the effects of work–family conflict and work–family facilitation on employee innovation in the digital era. Based on resource conservation theory, this study regards the work–family relationship as a conditional resource. Employees who are in a state of lack of resources caused by work–family conflict will maintain existing resources by avoiding the consumption of further resources to perform innovation activities; employees who are in a state of sufficient resources are more willing to invest existing resources to obtain more resources. In this study, 405 employees from enterprises in the Chinese provinces of Jiangsu, Anhui, Sichuan, and Guangdong, and in the municipality of Tianjin were selected as the research object. These enterprises are knowledge-based companies, and their employees frequently transfer knowledge at work. We collected questionnaires from the frontline employees of these companies. The results show that negative and positive emotions mediate the effect of work–family conflict and work–family facilitation on employee innovation. Moreover, work flexibility has a significant moderating effect on the mediating role of emotions between work–family facilitation and employee innovation behavior. In the digital era, when facing different work–family situations, employees need to pay attention to and dredge their negative emotions to avoid reducing their innovative behaviors due to self-abandonment; in parallel, they need to guide their positive emotions toward innovation, so as to promote their innovative consciousness and behavior. This paper expands the research perspective of employee innovation behavior.

## Introduction

New technologies such as Tencent meeting have popularized social media and offered new models of communication and collaboration within and between organizations (Barker, 2010). Employees can deliberately choose where and when they want to work: at the traditional office, at home, or practically anywhere else, anytime. The home office has thus become a new fashion. The boundary between work and non-work areas has become increasingly blurred (Derks et al., 2016). Social sustainability and sustainable social innovation have never been more important. Many scholars have focused on the convenience brought by the new technology, but we focus here on the confusion brought to employees by the new technology. However, the involvement of social media in employees’ families may lead to an imbalance between work and family life. New technology facilitates an ability to work late at home that is a double-edged sword (Dockery and Bawa, 2018). On the one hand, it provides flexibility and permeability, which can be useful to accommodate work and family demands. On the other hand, increased availability for work can increase workload and encroach upon family time (Allen et al., 2014; Barber and Santuzzi, 2014; Harris et al., 2015; Derks et al., 2016; Gadeyne et al., 2018; Ma and Turel, 2019; Yang et al., 2019).

Although scholars of human resource management and employment relationships have been discussing the impact of digital technology on human resource management, there are still many problems that have not been solved, such as the balance between work and family relationships (Allen et al., 2014). “Balance” between work and home lives is a much sought after but rarely achieved state of being. Work–family researchers have successfully encouraged organizations, families, and individuals to recognize the importance of tending to their needs for balance (Kim et al., 2015; Zhang et al., 2019; Wan et al., 2021). The interaction between work and family is a conditional resource (Hobfoll, 1989) that determines the employees’ energy resources (such as time) that in turn are the necessary condition for innovation activities. Employee innovation behavior consists of the generation, promotion, and realization of new ideas. This requires employees to invest a considerable amount of time and energy. Stimulating employee creativity is a way to achieve organizational innovation and human resource management (HRM) innovation (Jiang et al., 2012). The work–family interaction has two opposite articulations: a negative relationship, namely work–family conflict, and a positive relationship where the two systems promote each other, called work–family facilitation. Work–family facilitation implies that employees acquire skills at work that are conducive to the fulfillment of family responsibilities. In management practice, with the rapid development of mobile Internet technology and the widespread popularity of various mobile devices, the aging of China’s population, the launch of the two-children policy, and the increase in the proportion of families where both parents work, the pressure of employees’ work and family roles is increasing, and work–family conflict is becoming increasingly prominent (Oliveira et al., 2013; Matias and Recharte, 2020; Bernhardt and Bünning, 2021).

While scholars have started to research HRM and organizational innovation and creativity, the era of digitalization posts ever-rising challenges to both scholars and practitioners in developing and improving innovative and effective HRM models in this new era. In the era of digitalization, helping employees deal with work–family problems and promoting innovation performance is a common concern of organizations. However, in the field of organizational management, the work–family relationship has not received sufficient attention. Prior studies have explored the buffering effect of one of the multiple-choice work–family states in a single-stress situation but have not explored the buffering path from the perspective of resource consumption (Freire and Bettencourt, 2020; Chen et al., 2021; Yildiz et al., 2021). In order to expand the application of work flexibility to the impact of work–family conflict and work–family facilitation on employee innovation, this paper divides work flexibility into work–family conflict and work–family facilitation and then discusses the boundary role of work flexibility in detail.

In different work–family situations, employees will make different judgments based on their own resources, which will have an important impact on their behavioral decisions through positive and negative emotions (Judge et al., 2006; Speights et al., 2019). The conservation of resources theory provides a good theoretical framework to explain work–family conflict and work–family facilitation and their impact on innovation behavior. In other words, individuals always have the motivation to protect existing resources and acquire new resources (Mayo, 2008). When resources are insufficient or are depleted, individuals will experience a series of negative emotional reactions and then take corresponding actions to avoid further loss of their own resources (Hobfoll, 1989). Therefore, based on the conservation of resources theory, this paper will attempt to explain the logical relationship between resources, emotions, and behavior. In fact, there are several channels through which employees may obtain resources, such as the workplace and family. With the deep transformation of China’s human resources management system, the role of work flexibility in the workplace is becoming increasingly evident (Allen et al., 2013; Bayazit and Bayazit, 2017). In the enterprises that have implemented well-running work flexibility systems, employees can also obtain energy resources from the enterprise system (Srivastava, 2011). Based on this, can the energy resources provided by work flexibility affect the status of work–family conditional resources? If so, how does work flexibility affect the relationship between work–family status and employee innovation behavior? In relation to the current concern over the building of a harmonious work–family relationship, this problem is of great research value and should be addressed.

Therefore, what emotions are experienced in different work–family statuses, and how do they affect employees’ innovative behavior? From the perspective of resource consumption and supply, this paper discusses the mechanism of the impact of work–family conflict and work–family facilitation on employee innovation behavior. Based on the conservation of resources theory, this study explores the impact of resource status on employee innovation behavior, and the role of work flexibility and employee emotion. This study provides a new perspective for a comprehensive understanding of the relationship between work–family conflict/work–family facilitation and employee innovation behavior, as well as a useful reference for enterprises to intervene in employee innovation behavior from the perspective of work–family integrated management. Finally, this study further promotes the integration of relevant theories and achievements in the field of labor relations, human resources management systems, and organizational behavior, and provides a new theoretical perspective and practical guidance for promoting employees’ innovative behavior.

## Hypothesis Development

The connotation of conservation of resources theory must be based on the definition of resources. According to Hobfoll (1989), “resources refer to the material objects, conditions, personality characteristics, energy and other objects that individuals think are valuable and their corresponding acquisition methods.” These resources are specifically divided into material resources, conditional resources, personality traits, and energy resources. Conditional resources include marriage and family. As long as the conditions are valued and pursued, they are a type of resource. Energy resources, including time and so on, are not based on their intrinsic value, but on their contribution to obtaining other types of resources (Hobfoll, 1989; Bakker and Demerouti, 2007). The work–family relationship discussed in this paper is a summary of employees’ social relations, which is a type of conditional resource. The lack of this resource state also affects the energy resources of employees, that is, employees in the work–family conflict state spend time and energy (energy resources) to solve the conflict. The discretionary time brought by work flexibility can provide energy resources.

### Work–Family Conflict, Work–Family Facilitation, and Employee Innovation

The conservation of resources theory posits that when there are insufficient resources, people will strive to maintain their resources in order to effectively deal with the situation (Hobfoll, 1989; Bakker and Demerouti, 2007). Conversely, the accumulation of resources enables people to reinvest them in their work. When employees have sufficient resources and their investments can obtain good returns, they tend to invest “redundant” resources to obtain additional resources; on the contrary, when they are in a state of lack of resources, they will take actions to reduce their “surplus” and their further loss of resources (Hobfoll, 1989).

Work–family status can be divided into work–family conflict and work–family facilitation. When facing work–family conflict, employees will think that there is an imbalance between work and family, and this will generate a negative working environment (Zhang et al., 2012; Unruh et al., 2016). Employees will think that they cannot obtain and expend the same conditional and energy resources from both their organization and their family at the same time, so they perceive a lack of resources. Following this logic, we argue that work–family conflict is a state of conditional resource loss, in which employees will take actions to avoid the further loss of existing surplus resources. Innovation behavior mostly consists of initiative work behavior, which is not explicitly required by the organization (Parker and Collins, 2010). Employees facing conflict will think that they do not have enough resources to perform innovative activities and thus will not engage in initiative work behaviors, such as innovation, as a way to curb the further loss of resources. Studies have shown that work–family conflict, as a source of blocking stress, can reduce employees’ creativity (Innstrand et al., 2008; Luo, 2016). Specifically, first, the challenging characteristics of innovation behavior put forward higher requirements for individual resources investment (e.g., passion; Shan et al., 2019). However, work–family conflict will consume individual resources, and this can also lead to employee behavior that is detrimental to the organization (Yildiz et al., 2021). Individuals who suffer from resource loss tend to protect existing resources to avoid further losses, and thus, they will avoid being involved in innovation activities with a high demand for resources. Second, the risk characteristic of innovation behavior is also an important reason for the individual to participate in it. Work–family conflict transmits adverse environmental information to individuals such as conflict, oppression, and restraint, which increase the individual’s perception of external environmental risks, and thus reduce the possibility of their active participation in innovation (Lyu and Fan, 2020). Finally, the complexity of innovation behavior determines that individuals engaged in innovation need to expand their cognitive scope, constantly search for relevant knowledge and information, and form new ideas (Castaneda and Cuellar, 2020). However, individuals with work–family conflict focus on the resolution of conflicts and contradictions; this will reduce their cognitive attention to other aspects, which in turn is not conducive to the formation and development of innovative behavior. In this case, in order to preserve their surplus resources, employees reduce their work input, which may lead to a decline in the quality of their work and may make it more difficult for them to participate in innovation behavior. Therefore, as has been shown in previous studies, work–family conflict has a negative impact on knowledge workers’ innovative behavior (Jiayi et al., 2020).

Similarly, when employees experience work–family facilitation, they will acquire some skills because of their work, which is conducive to better performance in family responsibilities (Wayne et al., 2007). This state will provide employees with personal resources such as family support, time, and energy, so that they can feel positive about work, meet the needs of the workplace, reasonably handle family relations, and know that their contribution is meaningful (Chen and Chiu, 2009; Deng and Gao, 2017). According to the conservation of resources theory, individuals with more resources can use their existing resources to obtain even more resources, and this will encourage them to have a positive mental state and work behavior (Halbesleben and Wheeler, 2008). Work–family facilitation makes employees believe that they are in a state of sufficient resources, which in turn will promote their work identity and make them more inclined to invest “redundant” resources (including time, energy, etc.) to obtain more resources. In this case, the knowledge and skills acquired in the family can become the basis for the generation of new ideas at work, while the positive emotions experienced in the family can affect the employee’s willingness to innovate, thus promoting innovative behavior. Previous studies have shown that family support can promote employee innovation and that the family environment has an independent and significant effect on individual innovation behavior (Madjar et al., 2002; Zhang et al., 2019). As a result, employees facing work–family facilitation will regard work as an asset for sustainable development and family as a harbor for support, which is conducive to employees’ additional innovation activities. Based on this, this study proposes the following hypotheses:

*H1a*: Work–family conflict is negatively related to employees’ innovative behavior.

*H1b*: Work–family facilitation is positively related to employees’ innovative behavior.

### The Mediating Role of Employees’ Emotions

While facing a work–family dual state, employees will produce both negative and positive emotions due to conflicts and gains, which will have an important impact on subsequent behavioral responses (Barnes et al., 2015). Emotions may be divided into the positive and negative (Watson and Clark, 1984); they are physiological and psychological responses to good or bad information from the environment and depend on short-term or continuous evaluation. Peter (1990) and Folkman (2013) proposed the concept of work-related emotions. Negative emotions include depression, pain, worry, and tension, while positive emotions include ease, satisfaction, calm, optimism, and enthusiasm. These emotions are significantly related to employees’ work experience (Peter, 1990). Different work and family statuses, employees’ assessment of future development, and their own resources will stimulate different emotions, which will lead to corresponding behavioral decisions. Some studies have found that, when an individual evaluates a situation as a potential threat to their growth and future development, they will experience a negative emotion; on the contrary, when they evaluate it as a challenge and encouragement to their growth and future development, they will have a positive emotion (Folkman, 2008). The majority of existing studies argue that positive emotions are conducive to creativity, while negative emotions are not (Isen et al., 1987; Amabile et al., 2005).

When employees face work–family conflict, their innovation behavior is inhibited due to their lack of resources. Their response in terms of emotion or attitude is an important factor explaining the influence of work–family conflict on innovation behavior (Choi and Kim, 2012; Abstein and Spieth, 2014). According to the theory of resource conservation, when individual resources are threatened or lost, or the resources invested do not allow corresponding returns to be obtained, negative emotional experiences such as stress and anxiety will occur (Shantz et al., 2016). Work–family conflict is a state of lack of conditional resources, which brings pressure, conflict, and contradiction to employees (French and Allen, 2019). In this case, employees will think that they cannot obtain more resources from the organization or the family and that their time and energy (i.e., their energy resources) are consumed in solving the conflict. Employees will be prone to generate negative emotions and will enter a state of further lack of resources. This emotional state can affect their action orientation and behavioral intention (Barnes et al., 2015) and is an important factor affecting employee creativity (George and Zhou, 2002; Bledow et al., 2012). Work–family conflict entails a lack of resources. After the further consumption of energy resources, staff resources will be in shorter supply. Negative emotions will further deepen employees’ perception of resource shortage and will make employees feel that their own resources are decreasing, thus encouraging them to avoid innovation behavior. At the same time, studies have shown that negative emotions can reduce cognitive flexibility and inhibit individual creativity (Tests, 2018). Therefore, negative emotions may play a mediating role between work–family conflict and innovation behavior. When employees face work–family conflict and have negative emotions, this unpleasant emotional experience will stimulate then to take action to escape from this state. At the same time, in order to ease the negative emotions and reduce the further loss of resources, the innovative behavior of employees may be reduced.

Similarly, when employees face work–family facilitation, because they are in a state of sufficient resources, the acquisition and surplus of resources will enhance their well-being (Rappaport, 1981). According to the conservation of resources theory, individuals with more resources are not only less vulnerable to resource depletion but are also able to acquire new resources (Hobfoll, 2011), thus showing a more positive mental state and behavior (Halbesleben and Wheeler, 2008). Some scholars have established and broadened the model of positive emotions, arguing that positive emotions can broaden the focus of attention and the behavioral skills of individuals, so as to supplement individual social, intellectual, and physical resources (Fredrickson, 1998; Fredrickson et al., 2003; Waugh and Fredrickson, 2006). Work–family facilitation is a state of sufficient conditional resources, so that the skills learned by employees at work can be used to fulfill family responsibilities, and can generate goodwill and trust in the organization. In this case, employees’ evaluation of their work situation will be conducive to their own development, as they will experience a more positive emotional experience, which will promote the generation of positive emotions (Hill, 2005). Because positive emotions can help to increase positive behaviors and reduce negative behavioral tendencies, the positive emotions generated by the employees can further fill their resource status, so that they may make use of redundant resources for innovation. Research shows that individuals with positive emotions have a higher ability to classify concepts, higher efficiency in problem-solving, and higher creativity (Isen et al., 1987; Isen, 2005, 2012; Nelson and Sim, 2014). Positive emotions can also improve the cognitive flexibility of individuals, thus promoting individual creativity. Individuals with a positive mental state are more likely to shape good psychological and social resources, which are conducive to employees’ creative behavior (Tests, 2018). Therefore, when employees face work–family facilitation and have positive emotions, they are more likely to choose active behaviors in order to maintain that experience. This positive state can promote individual divergent thinking and creative problem-solving (Yang and Hung, 2014), which can promote innovation. Therefore, this study proposes the following hypotheses:

*H2a*: Negative emotions mediate the relationship between work–family conflict and innovation.

*H2b*: Positive emotions mediate the relationship between work–family facilitation and innovation.

### The Moderating Role of Work Flexibility

It is self-evident that the practice of human resources management has an impact on innovative behavior. In particular, the work flexibility system gives employees more time and space to conceive and practice new ideas. At the same time, it is conducive to the balance between work and leisure, thus improving efficiency (Dawkins and Simpson, 1994; Hansen et al., 2010; Dahm et al., 2015). From the perspective of resource consumption, work–family conflict, as an important source of individual pressure, will undoubtedly accelerate the loss of individual resources (Cole and Secret, 2012). From the perspective of resource supply, individuals who have experienced work–family conflict will usually actively seek external resources to reduce the harm brought by conflicts (Behson, 2002; Pattusamy and Jacob, 2016). The work flexibility system can provide emotional understanding and free time for individuals (i.e., energy resources), thus becoming an important way for individuals to augment their resource supply. Allen et al. (2013) showed that work flexibility is negatively correlated with work–family conflict. We hope to explore whether work flexibility regulates the relationship between work–family status and employees’ emotions, i.e., whether work flexibility weakens the positive relationship between work–family conflict and negative emotions and strengthens the relationship between work–family facilitation and positive emotions.

Specifically, first of all, work flexibility can effectively alleviate the impact of work–family conflict on employees’ negative emotions. It provides time resources that can alleviate the resource loss caused by work–family status and increase the resource reserve of employees. Unlike work–family conflict, which consumes resources, work flexibility provides energy support (i.e., time) to employees, which eliminates negative feelings from the process of work. For example, a work flexibility system gives employees more available time (Dahm et al., 2015). Employees can use this energy resource to solve work–family problems and to feed resources back into organizational innovation activities. The work flexibility system is implemented by the management of enterprises, and in this way, the function of human resources management practice is effectively brought into play. A humanized management system helps employees solve work–family conflicts and reduce their negative experiences of the work process (Vela-Jimenez et al., 2014; Martínez-Sánchez et al., 2019). Secondly, work flexibility can improve the impact of work–family facilitation on employees’ positive emotions. This is mainly because work flexibility provides employees with more energy resources, by giving them the right to go to/from work freely. It provides them more time so that they can freely arrange their working hours. As the resources provided by work flexibility can better meet the needs of employees for energy resources, strengthen the resource adequacy of work–family facilitation, increase the sense of resource acquisition, and promote the positive emotions of employees, employees will more actively use their surplus resources to actively participate in innovation and in other activities (Biron and van Veldhoven, 2012; Dahm et al., 2015).

When the work flexibility system can play an effective role, the energy resources of employees are at a high level, promoting the autonomy and enthusiasm of employees. On the contrary, when the work flexibility system is low, the energy resources’ employees can bring to bear to deal with company and family affairs are reduced, and work–family facilitation will change into a conflict state. In this case, due to the lack of work flexibility, employees cannot obtain enough energy resources when in a state of work–family conflict. Subjected to pressure for high performance, employees will be prone to have a negative work experience, such as feeling anxiety and emotional exhaustion, which will inhibit their innovative behavior. Due to the gain of work flexibility, employees will obtain more energy resources for work–family facilitation. The employees will reinvest their surplus resources and feed them back into the organization, improving employees’ innovative behavior. Therefore, this study proposes the following hypotheses:

*H3a*: The higher the level of work flexibility, the weaker the positive impact of work–family conflict on employees’ negative emotions.

*H3b*: The higher the level of work flexibility, the stronger the positive impact of work–family facilitation on employees’ positive emotions.

Based on the previous discussion, one can see that work flexibility regulates the relationship between work–family status and employee emotions and improves employees’ emotional experiences in the process of dealing with work–family problems (Shockley and Allen, 2007; Ollier-Malaterre, 2010). The work flexibility system, based on the position of employees, does not impose working hours on them. While ensuring that employees’ work flexibility does not damage the rights and interests of the enterprise, it also requires employees to perform creative activities, which can promote innovation (Broekaert et al., 2016; O’Rourke, 2021). Specifically, when employees are in a negative mood, the energy resources (i.e., time) provided by work flexibility can help them to relieve their negative emotions experienced in the process of work and avoid a situation in which they refuse to perform creative activities through other behaviors. On the contrary, when employees are in a positive mood, work flexibility can play a positive role, and employees more willingly engage in additional working hours (Dahm et al., 2015). This provides a basis to further strengthen the employees’ sense of resource acquisition and promote their innovative behavior. Therefore, work–family status and work flexibility can jointly affect employees’ emotional attitudes and behavior. In other words, work flexibility not only regulates the relationship between work–family status and positive and negative emotions but also further regulates the mediating role of positive/negative emotions between work–family facilitation/work–family conflict and innovative behaviors. Based on these considerations, we constructed a moderated mediation model. Specifically, positive (and negative) emotions mediate the influence of work–family status on innovation behavior, although the level of work flexibility will affect this mediating role. Therefore, this study proposes the following hypotheses:

*H4a*: Work flexibility moderates the mediating role of negative emotions between work–family conflict and innovation behavior, i.e., the higher the level of work flexibility, the stronger the mediating role of negative emotions.

*H4b*: Work flexibility moderates the mediating role of positive emotions between work–family facilitation and innovation behavior, i.e., the higher the level of work flexibility, the stronger the mediating role of positive emotions.

**Figure 1:**
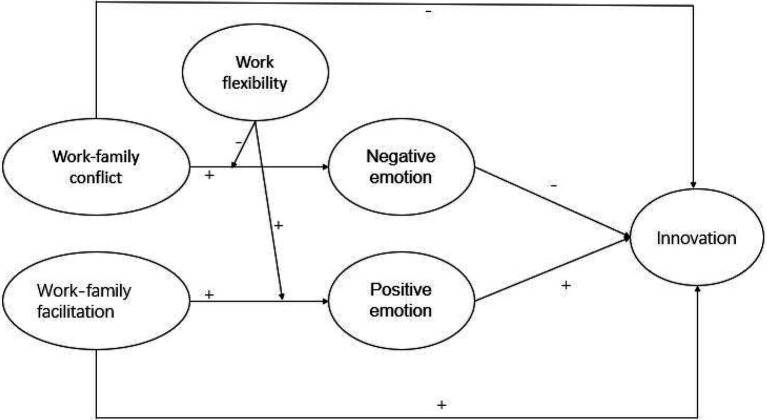
Theoretical model.

Our research model is shown in Figure 1.

## Research Methods

### Sample and Collection

We collected our data from multiple organizations in the provinces of Jiangsu, Anhui, Sichuan, and Guangdong, and in Tianjin City from December 2018 to March 2019. The local government helped us connect with local companies. The managers of the companies participating in the questionnaire survey have high performance and innovation requirements for employees. We asked employees to report their work–family facilitation, work–family conflict, work flexibility, and employee innovation. We measured work–family conflict, work–family facilitation, work flexibility, and positive and negative emotions at time 1. Two weeks later, we measured employees’ innovation behavior. Data collection was performed in two ways: (1) by e-mail, mainly through the enterprise contact person who sent the link to the electronic version of the questionnaire and the answer instructions to the respondents and (2) by on-the-spot recycling, in which members of the research group visited the enterprises, distributed questionnaires to the subjects, and collected them on the spot. A total of 518 questionnaires were collected in this survey. After eliminating the invalid questionnaires with missing answers and too many similar options, 405 valid questionnaires were obtained, with an effective recovery rate of 78.18%. The composition of the valid samples is shown in Table 1. It can be seen that the samples have a wide distribution and meet the basic requirements of the study. In terms of gender, 54.3% of the participants were male; 58.5% were aged 26–35; 63.7% were married; 55% had children; 58.7% held a bachelor’s degree or above; and 70.6% had worked for 1–5 years.

**Table 1:** Profile of the respondents.

Name	Category	Number	Percent	Name	Category	Number	Percentage
Gender	Male	220	54.3	Tenure	Up to 1 year	72	17.8
	Female	185	45.7		1–3 years	163	40.2
Age	25 years old and below	111	27.4		3–5 years	139	34.4
	26–35 years old	237	58.5		More than 5 years	31	7.6
	36–45 years old	50	12.3	Income	Below 2,000	1	0,2
	46 years old and above	7	1.7		2,001\u20134,000	138	34.1
Marriage	Unmarried	145	35.3		4,001\u20136,000	170	42
	Married	258	63.7		6,001\u20138,000	66	16.3
	Divorced	4	1		Over 8,000	30	7.4
Child	No child	182	44.9	Position level	Grassroots/Primary	342	84.4
	One child	186	45.9		Middle/Intermediate	58	14.3
	Two or more children	37	9.1		Senior/Junior	5	1.2
Education	High school or below	43	10.6	Work overtime	No	68	16.8
	Vocational school/College	124	30.6		Yes	337	83.2
	Undergraduate	214	52.8	Absence and early leave	No	323	79.8
	Master degree or above	24	5.9		Yes	82	20.2

### Measures

In this study, we selected mature scales available both at home and abroad, which have been widely used in China to ensure the reliability and effectiveness of the measurement variables. We translated these items from English into Chinese in accordance with the “translation and back translation” procedure. Except for the control variables, we used a 7-point Likert scale to measure all variables, ranging from 1 = *not compliant* to 7 = *conforming*.

In order to ensure the reliability of the results of the analysis of the sampled data, the Cronbach’s α coefficient was used to measure the internal consistency of each scale item. The calculated Cronbach’s alphas of work–family conflict, work–family facilitation, work flexibility, positive emotions, negative emotions, and employee innovation behavior were all greater than 0.7, indicating good reliability of the scales employed.

#### Work–Family Conflict

The scale used to measure work–family conflict was adapted from Grzywacz and Marks (2000). The scale consists of eight items, four of which measure work–family conflict and four of which measure work–family facilitation. A sample item is as follows: “Stress at home makes you irritable at work.” The Cronbach’s α coefficient for work–family conflict was 0.912.

#### Work–Family Facilitation

The scale used to measure work–family facilitation was adopted from Grzywacz and Marks (2000). A sample item is as follows: “Having a good day on your job makes you a better companion when you get home.” The Cronbach’s α coefficient for work–family facilitation was 0.884.

#### Work Flexibility

We selected and revised the work–family boundary flexibility scale developed by Matthews (Matthews and Barnes-Farrell, 2010) and formed a work flexibility load scale with three items. A sample item is as follows: “In order to meet my family and personal life responsibilities, I can go to work and leave work at any time.” A 7-point Likert design was adopted, with 1 = *total non-compliance* and 7 = *full compliance*. The Cronbach’s α coefficient for work flexibility was 0.916.

#### Positive and Negative Emotions

We modified the Positive and Negative Affect Scale (PANAS) developed by Watson et al. (1988). Our revised scale includes two emotional dimensions, i.e., positive emotions and negative emotions, for a total of 10 items. The positive emotions scale consists of five dimensions describing positive emotions, such as “happy” and “encouraged”; the higher the score of the positive emotion, the higher the level of the positive emotion at work. The negative emotions scale consists of five dimensions that describe negative emotions, such as “boredom” and “depression.” The higher the score, the stronger the emotion. After measurement, the Cronbach’s α coefficient of the positive emotions scale was 0.970, while that of the negative emotions scale was 0.963.

#### Innovation

We revised it according to Scott (Scott and Bruce, 1994). Our revised scale had 7 items, such as “I am willing to take the lead in trying new ideas or methods”; “I will seek new methods to solve problems”; and “I can generate new or breakthrough ideas.” A 7-point Likert scale was used to evaluate the innovation behavior, with the endpoints 1 = *totally disagree* and 7 = *completely agree*. The Cronbach’s α coefficient of this scale was 0.919.

#### Control Variables

Considering that demographic variables may affect employees’ innovative behavior, we controlled gender, age, marriage, children, education, tenure, income, and position level (Tang et al., 2017).

## Data Analysis and Results

### Common Method Bias Test

Common method variance (CMV) refers to the artificial variation among variables caused by use of the same subjects or data sources, similar measurement situations, common project context, or project characteristics (Podsakoff et al., 2003). Although in this study we used a two time-point data collection method to control the common method variance problem, the fact that the items in each questionnaire were filled in by one person means that there could still be common method variance in the measurement process. In this study, Harman’s single factor test was used to test the degree of variation of the sample data. Six factors were extracted through principal component analysis. The results show that the variance explained by the first factor was 38%, i.e., less than the critical value of 40%. This indicates that the CMV of the data employed in this paper was not significant, although this issue deserves further investigation.

### Confirmatory Factor Analysis

In this study, the software Amos 24 was used to conduct confirmatory factor analysis on six variables (i.e., work–family conflict, work–family facilitation, work flexibility, positive emotions, negative emotions, and employee innovation behavior) to test the discriminant validity of the measurement variables (see Table 2). It can be seen from Table 2 that the six-factor model is the most suitable (*χ*^2^/df = 3.301; NFI = 0.911; TLI = 0.927; CFI = 0.936; RMSEA = 0.075), as it clearly performs better than the other models, indicating that the measurement variables in this study have good discriminant validity. One can see from Table 2 that the six-factor model has the best fit compared to other models, and each fitting index is at an acceptable level, indicating that the six main constructs in this study have good discriminative validity.

**Table 2:** Confirmatory factor analysis.

Model	Model factor	$\chi^{2}$	df	$\chi^{2}$ / df	NFI	RFI	IFI	TLI	CFI	RMSEA
Single factor	A + B + C + D + E + F	7,435.584	350	21.245	0.385	0.287	0.397	0.297	0.394	0.224
Two factors	A + B + C + D + E, F	6,359.977	349	18.223	0.474	0.43	0.488	0.444	0.487	0.206
Three factors	A + B + C, D + E, F	5,154.494	347	14.854	0.574	0.501	0.591	0.519	0.589	0.185
Four factors	A + B, C, D + E, F	4,638.253	344	13.483	0.616	0.579	0.635	0.597	0.633	0.176
Five factors	A + B, C, D, E, F	2,421.398	340	7.122	0.8	0.777	0.823	0.802	0.822	0.123
Six factors	A, B, C, D, E, F	1,023.391	310	3.301	0.911	0.899	0.936	0.927	0.936	0.075

*A=WFC, work–family conflict; B=WFF, work-family facilitation; C=WF, work flexibility; D=PE, positive emotions; E = NE, negative emotions; F:I,innovation*.

Because the survey data of this study were filled in by the employees themselves, it was necessary to conduct a common method variance test. Using Harman’s single factor test method, all measurement items were included in a common factor for model fitting (see the single-factor model in Table 2). It can be seen from Table 2 that the single factor model fitting is poor, which indicates that the CMV of the questionnaire data in this study is not significant.

### Statistical Description

In this study, gender, age, marriage, and whether to raise children were included as control variables. Analysis showed that these control variables had no significant effect on the dependent variables. The mean value, standard deviation, and correlation coefficient of each variable are shown in Table 3, with the square root of the average variance extracted (AVE) on the diagonal. According to Table 3, work–family conflict was negatively correlated with innovation behavior (*r* = −0.274, *p* < 0.05), while work–family facilitation was positively correlated with innovation behavior (*r* = 0.303, *p* < 0.05). There was a significant positive correlation between work–family conflict and negative emotions (*r* = 0.537, *p* < 0.05), and between work–family facilitation and positive emotions (*r* = 0.339, *p* < 0.05). Positive emotions were positively correlated with innovation behavior (*r* = 0.553, *p* < 0.05), while negative emotions were negatively correlated with innovation behavior (*r* = −0.329, *p* < 0.05). The results show that work–family conflict, work–family facilitation, work flexibility, negative emotions, positive emotions, and employee innovation behavior were significantly correlated at a moderate level, which allowed us to further perform a regression model test. It can also be seen from Table 3 that the critical values of the correlation levels were not higher than 0.75. Therefore, there was no serious multicollinearity problem in the analysis of the data.

**Table 3:** Descriptive statistics and correlation coefficients of the variables.

	1	2	3	4	5	6
WFC	**0.89**					
WFF	−0.165^**^	**0.87**				
WF	−0.708^**^	0.07	**0.92**			
PE	−0.415^**^	0.339^**^	0.390^**^	**0.94**		
NE	0.537^**^	−0.122^*^	−0.503^**^	−0.459^**^	**0.93**	
I	−0.274^**^	0.303^**^	0.210^**^	0.553^**^	−0.329^**^	**0.82**
M	2.98	4.18	4.53	5.15	2.36	5.24
SD	1.54	1.48	1.61	1.32	1.43	1.06

*indicates a significant correlation at 0.05 level (double tail); **indicates a significant correlation at 0.01 level (double tail); Bold indicates a square root of the average variance extracted (AVE). n = 405.WFC, Work–family conflict; WFF, work-family facilitation; WF, work flexibility; PE, positive emotions; NE, negative emotions; I, innovation behavior.

### Hypothesis Tests

In this study, we used the process macro program of SPSS 23 and the bootstrap method to test the hypotheses (Preacher et al., 2007). This method is superior to more traditional methods because it does not require a normal sampling distribution but can instead use the ordinary least squares regression to estimate the direct and indirect effects of the mediations and can use the 1,000 bias-correction guidance.

#### Main Effect Tests

The results of the main effect analysis of work–family conflict and work–family facilitation are shown in Table 4. According to Table 4, work–family conflict can significantly inhibit employee innovation behavior (*β* = −0.158, *p* < 0.001), while work–family facilitation can significantly increase employee innovation behavior (*β* = 0.189, *p* < 0.001). Thus, H1a and H1b are supported. In addition, it can be seen from Table 4 that work–family conflict can significantly increase employees’ negative emotions (*β* = 0.494, *p* < 0.001) and can also significantly reduce employees’ positive emotions (*β* = −0.315, *p* < 0.001); conversely, work–family facilitation can significantly increase employees’ positive emotions (*β*=0.247, *p* < 0.001), but has no significant effect on negative emotions (*β* = −0.033, *p* > 0.05).

**Table 4:** Standardized results of the main effects of the work-family status.

Variable	Innovative behavior of employees		Positive emotions		Negative emotions	
	Coefficient	Standard error	Coefficient	Standard error	Coefficient	Standard error
WFC	−0.158^***^	0.032	−0.315^***^	0.038	0.494^***^	0.040
WFF	0.189^***^	0.033	0.247^***^	0.039	−0.033	0.041

*** = p < 0.001; WFC, Work–family conflict; WFF, work-family facilitation.

#### Mediating Effect of Emotion

First, the process macro program in SPSS 23.0 and the bootstrap method were used to test the mediating role of employees’ emotions between work–family state and innovative behavior (see Table 5). It can be seen from Table 5 that the value of the mediating role of negative emotions between work–family conflict and employee innovation behavior was −0.0943; moreover, the 95% confidence interval of bootstrap = 5,000 (−0.1572, −0.0445) did not contain 0, thus indicating that the mediating role was significant. Thus, H2a is supported. The value of the mediating role of positive emotions between work–family facilitation and employee innovation behavior was 0.1233. The 95% confidence interval of bootstrap = 5,000 (0.0803, 0.1699) did not contain 0, thus indicating that the mediating role was significant. Thus, H2b is supported. In addition, we also found that the value of the mediating role of negative emotions between work–family facilitation and employee innovation behavior was 0.0258, and the 95% confidence interval of bootstrap = 5,000 was (0.0036, 0.0567), which indicates that the mediating role was significant. The value of the mediating role of positive emotions between work–family conflict and employee innovation behavior was −0.1513. The 95% confidence interval of bootstrap = 5,000 (−0.2029, −0.1128) did not contain 0, thus indicating that the mediating role was significant.

**Table 5:** Test results of the effect of employees’ emotions (n = 405).

Intermediate variable path	Mediating role value		Confidence interval (95%)	
	Coefficient	Standard error	BootLLCI	BootULCI
WFC → NE → I	−0.0943	0.0282	−0.1572	−0.0445
WFC → PE → I	−0.1513	0.023	−0.2029	−0.1128
WFF → NE → I	0.0258	0.0133	0.0036	0.0567
WFF → PE → I	0.1233	0.0229	0.0803	0.1699

*WFC, Work–family conflict; WFF, work-family facilitation; PE, positive emotions; NE, negative emotions; I, innovation behavior*.

#### Moderating Effect of Work Flexibility.

In this study, we used the macro program in SPSS 23.0 to test the moderating effect of work flexibility on work–family conflict/work–family facilitation and emotions (see Table 6). According to Table 6, the regulatory effect of work flexibility on the path from work–family conflict to negative emotions was not significant (*β* = −0.0024, *p* > 0.05); this indicates that work flexibility has a positive regulatory effect between work–family conflict and negative emotions; this result is contrary to H3a. Work flexibility had a significant moderating effect on the path from work–family facilitation to positive emotion (*β*=0.0444, *p* = 0.0094), indicating that work flexibility has a positive regulatory effect between work–family facilitation and positive emotions. Thus, H3b is supported.

**Table 6:** Test results of the adjustment effect of work flexibility (n = 405).

Adjustment term	Positive emotions		Negative emotions	
	Coefficient	Standard error	Coefficient	Standard error
WFC × WF			−0.0024	0.087
WFF × WF	0.0444^*^	0.017		

*WFC, Work–family conflict; WFF, work-family facilitation; WF, work flexibility*.

**Figure 2:**
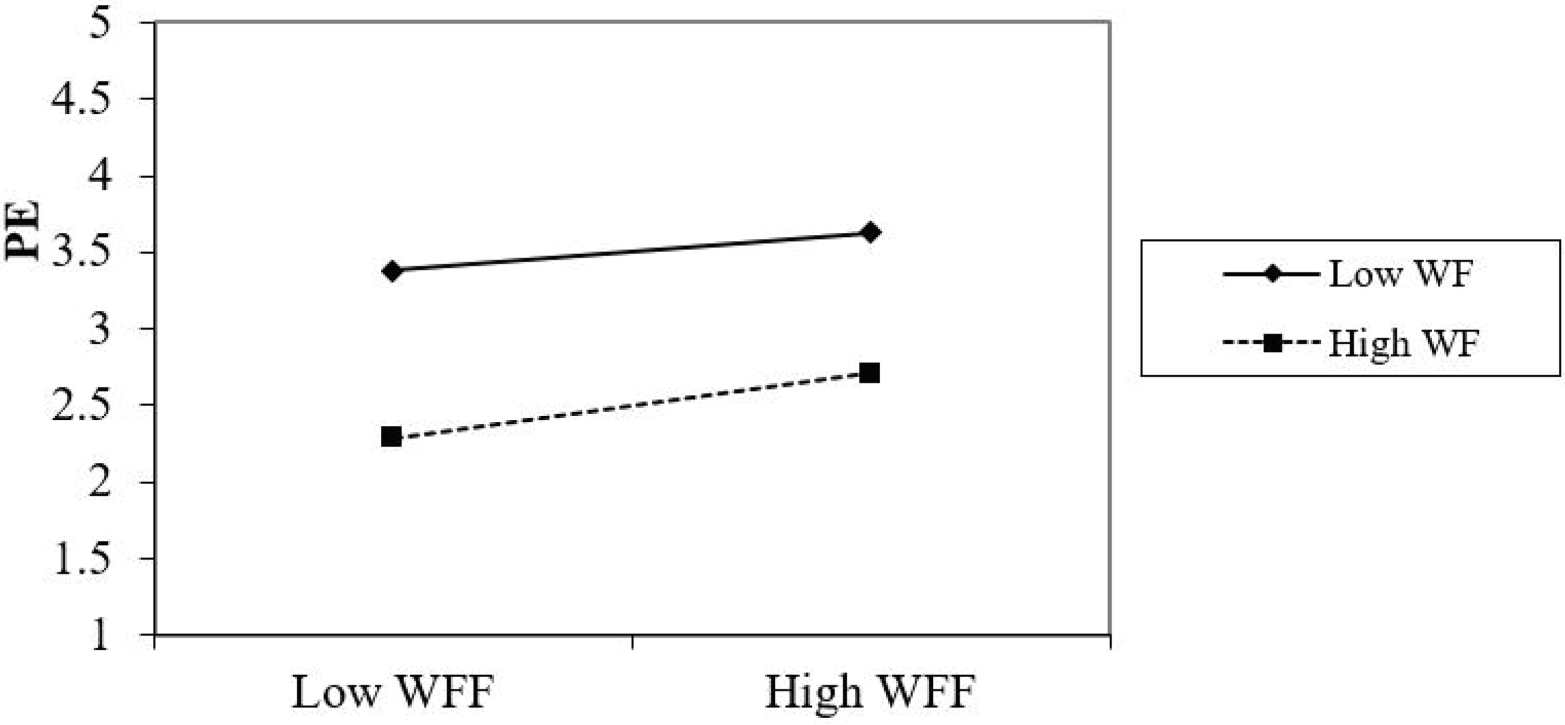
The moderating role of work flexibility. WFF, work–family facilitation; WF, work flexibility; PE, positive emotions.

In order to understand the essence of the regulation effect between work–family facilitation and positive emotions more clearly, all the samples were divided into two groups depending on work flexibility. In more detail, we considered the samples with low work flexibility to be those with values of work flexibility lower than the mean value minus the standard deviation, and the samples with high work flexibility were defined as those having values of work flexibility higher than the mean value plus the standard deviation. Then, the simple slope test and the simple effect analysis chart were drawn (see Figure 2). It can be seen from Figure 2 that work–family facilitation has a significant positive predictive effect on positive emotions when work flexibility is low, and a weakened predictive effect when work flexibility is high. This shows that work flexibility has a moderating role in this process, and hypothesis 3b is supported.

According to our analysis, work flexibility has a significant regulatory effect on positive emotions in work–family facilitation, but not on negative emotions in work–family conflict. Thus, hypothesis 4a is not supported. Based on this, we further verified the mediating role of work flexibility on positive emotions between work–family facilitation and innovation behavior (see Table 7). As can be seen from Table 7, the 95% confidence interval of the difference of indirect effects did not include 0, indicating that the difference in the indirect effects was significant. Thus, hypothesis 4b is supported.

**Table 7:** Bootstrap analysis of the moderated mediating role (5,000 samples).

Intermediate variable path	Index	Effect value	BootSE	Confidence interval (95%)	
				BootLLCI	BootULCI
WFF → PE → I	Eff1 (M-1SD)	0.0995	0.023	0.0518	0.1412
	Eff2 (M)	0.1266	0.0277	0.0706	0.1774
	Eff3 (M + 1SD)	0.1536	0.0372	0.0821	0.2242

*eff1/eff2/eff3 refer to a standard deviation below/equal to/higher than the mean value, respectively. WFF, work-family facilitation; WF, work flexibility; PE, positive emotions; I, innovation behavior*.

## Discussion

### Theoretical Implications

The theoretical contributions of this study are as follows. First, this paper investigated the two resource states of work–family conflict and work–family facilitation. Prior studies have explored the impact of work–family status on employees’ innovation activities from a single perspective of either work–family conflict or work–family facilitation (Madjar et al., 2002; Halbesleben and Wheeler, 2008; Chen and Chiu, 2009; Wang et al., 2012; Yang and Hung, 2014; Jiayi et al., 2020). This study examined the relationship between work–family status and innovation and verified that there are differences in the impact of work–family conflict and facilitation on the innovation behavior of employees, thereby supplementing the literature on the work–family relationship. In fact, this study found that the impacts of work–family conflict and facilitation on innovation behavior are diametrically opposite. Therefore, the conclusions of this study enrich the current literature on the work–family state.

Second, the mediating role of employees’ emotions between the work–family state and innovation behavior was confirmed. It is believed that individuals will have different emotions when facing different resource states, which will affect their innovation behavior. Moreover, work flexibility plays a moderating role between work–family facilitation and positive emotions, but not between work–family conflict and negative emotions. As a supplement to energy resources, work flexibility can regulate the relationship between work–family state and emotions. Therefore, this paper also demonstrates the regulation mechanism between work–family state and emotion.

Third, this study expanded the conservation of resources theory. Prior studies have examined work–family status from the perspective of resource consumption (Hobfoll, 1989; Innstrand et al., 2008; Halbesleben and Wheeler, 2008; Jiayi et al., 2020). On this basis, we used the perspectives of resource consumption and resource supply to verify the relationship between work–family state, emotions, and innovation. In this way, we could consider not only the pressure on employees when they face a resource shortage (work–family conflict) but also the role of resource supplement. In the resource shortage state, employees will use energy resources as a supplement of conditional resources, so as to reduce the loss of resources. In order to conserve resources, employees will avoid innovative activities that require additional energy resources (i.e., time, knowledge, and energy). To a certain extent, this verifies the feasibility of the work flexibility system in supplementing the energy resources available to employees, so as to achieve innovation.

### Practical Implications

Our findings have also some implications for management practice. First, organizations should take measures to improve the work–family relationship and implement family-friendly policies, which can improve the innovation performance of enterprises. Since it was first suggested that social factors affect creativity (Abstein and Spieth, 2014), both Chinese and foreign scholars of the antecedents of creativity have begun to turn their attention toward social relations, although few of them have studied family relations (Chen et al., 2009). If enterprises want to stimulate employees’ innovative behavior, they must make efforts in several ways and attach importance to the role of employees’ families (Wang et al., 2012). For example, family harmony and family attitude toward their careers should be taken as selection criteria when employees enter the job. Family should be included in the team building work of the enterprise after the employee enters the job, as this not only allows employees to develop recognition of their own work but also strives for the identity of the employee’s family for the occupation. Employees should be allowed to perform family responsibilities, take holidays, and have work flexibility arrangements; this will help employees reduce their family responsibilities (Kreiner et al., 2009).

Second, from the perspective of employees’ emotions, this study provided a new explanation path for the mechanism between work–family state and innovation behavior. When employees face different work–family statuses, they need to pay attention to, and dredge, their negative emotions to avoid giving up their innovative behavior due to self-abandonment; in parallel, they also need to guide their positive emotions in an innovative way to promote their innovative awareness and behavior (Meng and Meng, 2018). If employees have negative emotions in the process of work due to conflict between work and family, and the communication is not very effective, managers should ease the negative emotions of employees in a timely fashion and prevent employees from relieving them through other behaviors. Specifically, in terms of resources, we should supplement the resource consumption of employees in the process of work in a timely fashion, and reduce the emotional problems caused by the lack of resources. In terms of life, we should understand the difficulties of employees’ families in a timely fashion. For example, to ensure the work–family balance of employees, enterprises can effectively negotiate with them (Baltes et al., 1999), so as to obtain beneficial results for both sides, and at the same time let employees feel the warmth of their enterprise, so as to reduce the emotional problems experienced by employees in the work process.

Third, when designing jobs, organizations should take full account of work flexibility, such as flexible schedule, compressed workweek, and telecommuting, so that employees can obtain a higher degree of work freedom, handle the work–family relationship, and engage in an independent innovation behavior. This study integrated the work domain and the family domain and verified the role of work flexibility in overcoming work–family conflict. We found that work flexibility can improve employees’ work experience and reduce negative emotions by supplementing employees’ time and energy resources. As such, this study provides a reference for the design of the enterprise system. Moreover, work flexibility arrangements can help employees better allocate their time and energy. When employees face the work–family relationship, and especially when there are conflicts, they may not fully understand the relevant human resources management practices of the organization based on their own level and thus adopt some exclusion behaviors. In this case, the work flexibility system can communicate with them through its own role, as a milder and more acceptable work system, to help employees better handle the work–family relationship and effectively promote the implementation of innovation activities.

### Limitations and Future Research

This study has some limitations. First, the survey data came directly from the employees. In view of this, in terms of employees’ behavior, follow-up research can measure the subjectively perceived employees’ innovation behavior and compare it with the employee’s perceived innovation behavior, to better understand the degree of employee’s innovative behavior more intuitively. In addition, although our research group repeatedly emphasized the confidentiality and the academic value of our questionnaire, there is still the possibility that employees were unwilling to report their actual situation regarding their work–family status. Therefore, follow-up research can reduce the research error as much as possible according to the actual situation and in the form of other reviews.

Second, this study focused only on the moderating role of the work flexibility system, i.e., a situational factor, in the influence of the work–family state on employees’ emotions. However, in fact, the work flexibility system can also improve employees’ innovative behavior. In future research, we can further compare and explore the difference between work flexibility and work–family state in the process of influencing employees’ innovative behavior.

Third, based on our general research question, the present study clarified the impact and mechanisms of the work–family state on employee innovation; as such, the impact and process of the work–family state on employees’ innovation behavior remain to be clarified. Further in-depth and targeted research can be performed targeting different types of enterprises.

Finally, employees are forced to work from home amid the ongoing COVID-19 pandemic. This increases the working hours of employees at home and may also lead to an imbalance in work–family status, which is a topic for further research.

## Conclusion

The implementation of innovation-driven development strategies in China requires enterprises to construct new models of human resource management (HRM) to face increasing challenges and rapid changes in the digital era. Based on the resource conservation theory, this study explored the relationship between work–family state, employees’ emotions, and employee innovation behavior, and tested the regulatory mechanism in the context of work flexibility. The main findings of this study are as follows. First, work–family conflict can significantly inhibit innovation behavior, while work–family facilitation can significantly increase it. Second, negative emotions mediate between work–family conflict and innovation behavior, while positive emotions mediate between work–family facilitation and innovation behavior. Third, work flexibility plays a moderating role between work–family facilitation and positive emotions, but the moderating effect between work–family conflict and negative emotions is not significant. At the same time, work flexibility moderates the mediating role of positive emotions between work–family facilitation and innovation behavior, i.e., the higher the level of work flexibility, the stronger the mediating role of positive emotions.

## Data Availability Statement

The raw data supporting the conclusions of this article will be made available by the authors, without undue reservation.

## Author Contributions

ZW, XQ, and YJ performed conceptualization and methodology. ZW and XQ done formal analysis and writing—original draft preparation. ZW and XZ investigated the study. ZW and YJ performed supervision. ZW done funding acquisition. All authors have read and agreed to the published version of the manuscript.

## Funding

This research was funded by National Natural Science Foundation of China (Nos. 71862013, 71832007, 72162023, 71762016 and 71862019), and China Postdoctoral Science Foundation (No. 2018M642216).

## Conflict of Interest

The authors declare that the research was conducted in the absence of any commercial or financial relationships that could be construed as a potential conflict of interest.

## Publisher’s Note

All claims expressed in this article are solely those of the authors and do not necessarily represent those of their affiliated organizations, or those of the publisher, the editors and the reviewers. Any product that may be evaluated in this article, or claim that may be made by its manufacturer, is not guaranteed or endorsed by the publisher.
